# A magnet attached to the forehead disrupts magnetic compass orientation in a migratory songbird

**DOI:** 10.1242/jeb.243337

**Published:** 2021-11-18

**Authors:** Florian Packmor, Dmitry Kishkinev, Flora Bittermann, Barbara Kofler, Clara Machowetz, Thomas Zechmeister, Lucinda C. Zawadzki, Tim Guilford, Richard A. Holland

**Affiliations:** 1 School of Natural Sciences, Bangor University, Bangor LL57 2UW, UK; 2 Institute of Avian Research ‘Vogelwarte Helgoland’, Wilhelmshaven 26386, Germany; 3 School of Life Sciences, Keele University, Newcastle-under-Lyme ST5 5BG, UK; 4 Biological Station Lake Neusiedl, Illmitz 7142, Austria; 5 Nationalpark Neusiedler See – Seewinkel, Apetlon 7143, Austria; 6 Austrian Ornithological Centre, Konrad-Lorenz Institute of Ethology, University of Veterinary Medicine Vienna, 1160 Wien, Austria; 7 Department of Zoology, Oxford University, Oxford OX1 3SZ, UK

**Keywords:** Celestial compass, Eurasian reed warbler, Environmental cue, Orientation cage, Navigation, Migration, Orientation, Songbird, Star compass

## Abstract

For studies on magnetic compass orientation and navigation performance in small bird species, controlled experiments with orientation cages inside an electromagnetic coil system are the most prominent methodological paradigm. These are, however, not applicable when studying larger bird species and/or orientation behaviour during free flight. For this, researchers have followed a very different approach, attaching small magnets to birds, with the intention of depriving them of access to meaningful magnetic information. Unfortunately, results from studies using this approach appear rather inconsistent. As these are based on experiments with birds under free-flight conditions, which usually do not allow exclusion of other potential orientation cues, an assessment of the overall efficacy of this approach is difficult to conduct. Here, we directly tested the efficacy of small magnets for temporarily disrupting magnetic compass orientation in small migratory songbirds using orientation cages under controlled experimental conditions. We found that birds which have access to the Earth's magnetic field as their sole orientation cue show a general orientation towards their seasonally appropriate migratory direction. When carrying magnets on their forehead under these conditions, the same birds become disoriented. However, under changed conditions that allow birds access to other (i.e. celestial) orientation cues, any disruptive effect of the magnets they carry appears obscured. Our results provide clear evidence for the efficacy of the magnet approach for temporarily disrupting magnetic compass orientation in birds, but also reveal its limitations for application in experiments under free-flight conditions.

## Abstract (precis)

**Summary:** Orientation tests in a small migratory songbird provide clear evidence for the efficacy of the magnet approach for temporarily disrupting magnetic compass orientation, but also reveal its limitations in experiments under free-flight conditions.

## INTRODUCTION

Birds are amongst the most mobile animals, with many species ranging over thousands of kilometres and between continents during their year-round movements. Such a mobile lifestyle requires the ability to orient and navigate reliably over long distances using positional (i.e. map) and directional (i.e. compass) information derived from environmental cues (e.g. Kramer, 1953, 1957). There is evidence for the use of at least three different compass systems for choosing and maintaining specific directions in birds (Chernetsov, 2017). Two of the three systems are based on celestial cues: the time-dependent sun compass, which requires the birds’ internal clock as reference (e.g. Kramer, 1949; Schmidt-Koenig, 1958), and the time-independent star compass (e.g. Emlen, 1967a,b; Mouritsen and Larsen, 2001; Pakhomov et al., 2017). The third system is the magnetic compass, which uses Earth's magnetic field (e.g. Merkel and Wiltschko, 1965; Wiltschko, 1968).

Since its first description in the 1960s, magnetic compass orientation in birds has received particular attention in many subsequent studies. It was shown that birds use the inclination (dip angle) of Earth's magnetic field rather than its polarity for orientation (Wiltschko and Wiltschko, 1972). Further, there is experimental evidence suggesting that in birds, magnetic compass orientation depends on the wavelength and the intensity of the available light (Wiltschko et al., 1993; Wiltschko and Wiltschko, 1995b; Muheim et al., 2002). The sensory basis for magnetic compass orientation, however, is still not fully resolved and is the subject of intensive research (e.g. Mouritsen, 2015, 2018; Nordmann et al., 2017).

A large number of experiments on avian magnetic senses, magnetic compass orientation and navigation have used migratory songbirds as a model for birds in general. If kept in captivity, migratory songbirds typically express increased locomotor activity (wing whirring and/or hopping) during periods normally used for their migratory flights – a behaviour referred to as migratory restlessness (or ‘Zugunruhe’; e.g. Berthold et al., 2000). As migratory restlessness is commonly concentrated towards the birds’ preferred migratory flight direction (Kramer, 1949), it can be used as a proxy for their orientation behaviour that is studied by means of small orientation cages (e.g. Emlen funnels; Emlen and Emlen, 1966) under controlled experimental conditions. When placed inside an electromagnetic coil system (e.g. Helmholtz coil system), such orientation cages allow assessment of the effect of specifically altered magnetic fields on birds’ orientation behaviour (e.g. Wiltschko, 1968). To date, the combined use of electromagnetic coil systems with orientation cages represents the most prominent methodological paradigm for studies on magnetic compass orientation and navigation performance in small bird species, especially songbirds.

However, when studying magnetic compass orientation and navigation performance in larger bird species and in the context of their behaviours during free flight, any precise alteration of the magnetic field birds experience is challenging, if not impossible. Instead, researchers have tried to assess birds’ use of Earth's magnetic field for orientation and navigation by depriving them of access to meaningful magnetic information. The most common approach for this is releasing birds with small magnets attached to their bodies (e.g. to the head or back), which was first suggested by physicist C. Maurain in 1926 (see Wiltschko and Wiltschko, 1995a, for a review). Permanent magnets such as the widely used rare-earth magnets (neodymium and samarium–cobalt magnets) produce strong magnetic fields which exceed Earth's magnetic field in total magnetic intensity (total field strength) within a radius of several centimetres. When attached close to birds’ putative magnetic receptors, such magnets should strongly interfere with Earth's magnetic field around them, leading to altered resultant magnetic fields in which the resultant vectors are forced to remain within a certain sector relative to the alignment of the magnets when the birds turn or move (e.g. Mouritsen et al., 2003). Such resultant magnetic fields are generally assumed to be uninterpretable and, thus, useless for orientation and navigation purposes (e.g. Mouritsen et al., 2003). Others, however, have questioned the efficacy of this approach (e.g. Wang et al., 2006; Nimpf et al., 2019).

Results from previous studies using magnets for disrupting magnetic compass orientation and navigation in birds appear rather inconsistent. Whereas some studies report a disruptive effect, suggesting the use of magnetic information, others found no such effect (see Wiltschko and Wiltschko, 1995a, for a review), or effects that varied between repeated experiments and between years (Ranvaud et al., 1991; Moore, 1988). Studies in which the birds apparently had access to other orientation cues (e.g. the sun) during the experiment tended to find no effect of the magnets on orientation and overall navigation performance (e.g. Wiltschko and Wiltschko, 1995a, and references therein; Mouritsen et al., 2003; Bonadonna et al., 2005; Gagliardo et al., 2013; Pollonara et al., 2015; Padget et al., 2017; but see Southern, 1972). This is generally inconclusive, as the lack of control over other orientation cues makes it difficult to rule out that Earth's magnetic field is actually used to obtain positional (i.e. map) and/or directional (i.e. compass) information under natural conditions. Beyond that, the efficacy of the specific treatment cannot be assessed without any preceding tests under controlled experimental conditions that allow no reversion to other orientation cues. Most of the earlier studies used magnets on pigeons (*Columba livia* f. *domestica*) during homing experiments (see Wiltschko and Wiltschko, 1995a, for a review). More recent studies that combined magnets with satellite telemetry to study bird behaviour on a large spatial scale mainly focused on the navigation performance of seabirds such as tubenoses (Procellariiformes) during foraging and homing flights towards their breeding colonies (e.g. Mouritsen et al., 2003; Bonadonna et al., 2005; Gagliardo et al., 2013; Pollonara et al., 2015; Padget et al., 2017). To date, however, there appears to be only one previous study using magnets on a songbird species (the barn swallow, *Hirundo rustica*, during homing experiments; Bochenski et al., 1960), despite songbirds being the most extensively studied taxon with regard to magnetic compass orientation and navigation. Further, to the best of our knowledge, there is not a single study applying the magnet approach in the context of compass orientation or navigation during seasonal migration.

Here, we investigated the effect of small magnets attached to the foreheads of first-year migratory songbirds, which are known to use Earth's magnetic field for setting their migratory direction. Birds’ orientation behaviour was repeatedly tested during their first autumn migration season, using orientation cages set up in the field. We expected that birds which have access to the Earth's magnetic field as their sole orientation cue would be generally oriented towards the seasonally appropriate natural migratory direction. When carrying magnets on their forehead under these circumstances, we expected the same birds to become disoriented as a result of the disruptive effect on their magnetic compass. When the birds are allowed access to other orientation cues (i.e. celestial cues) during the tests, however, we expected any disruptive effect of the magnets to be obscured, as the birds would probably fall back on another compass system (i.e. the star compass) for successful orientation. Our study, for the first time, tests the efficacy of magnets for disrupting magnetic compass orientation in songbirds under controlled experimental conditions.

## MATERIALS AND METHODS

### Study species and site

We selected the Eurasian reed warbler, *Acrocephalus scirpaceus* (Hermann 1804) (hereafter reed warbler), as our study species, because it represents a well-established model for studying magnetic compass orientation and navigation in songbirds and birds in general. Reed warblers are common long-distance migrants breeding in reed-lined habitats across a large part of Europe to western Asia and overwintering in sub-Saharan Africa (del Hoyo et al., 2006). Fieldwork for this study took place at Lake Neusiedl (Neusiedler See, in Austria, or Fertő, in Hungary), a shallow steppe lake situated at the north-western edge of the Pannonian Basin straddling the Austrian–Hungarian border. Experiments were performed at the Biological Station Lake Neusiedl in Illmitz, Burgenland, south-eastern Austria (47°46′08.9″N, 16°45′57.2″E).

### Assessment of the natural migratory direction

We obtained bird ring recovery data of reed warblers ringed at Lake Neusiedl during previous years, both from the Austrian Ornithological Centre (AOC) and the Hungarian Bird Ringing Centre (MME). These data were used to assess the natural migratory direction of reed warblers from the study population during autumn migration. We filtered the data for autumn recoveries (September–November) of birds that were ringed during the breeding and early migratory period (late May–August) of the same year and that were found at a distance of >250 km from the ringing site. The rationale for the latter was to avoid any directional bias due to a disproportionate number of recoveries from nearby ringing sites.

### Orientation experiments

#### Ethical statement

All applicable international, national and/or institutional guidelines for the care and use of animals were followed. The experiments were conducted in accordance with the national animal welfare legislation of Austria and with permission of the state of Burgenland (Abteilung 4 – Ländliche Entwicklung, Agrarwesen und Naturschutz; permit: A4/NN.AB-10216-7-2019). Additionally, the experiments received local ethical approval by the animal welfare ethics review body (AWERB) of Bangor University, where the corresponding authors (F.P. and R.A.H.) were employed during the period of data collection.

#### Capture and husbandry

For our two orientation experiments (experiment 1 in 2019 and experiment 2 in 2020), we captured reed warblers in the reed beds near the Biological Station during early September, i.e. during the species-specific autumn migration season. The birds were captured with mist nets as part of the Biological Station's bird monitoring and ringing project. Following capture and standard ringing procedures, we transferred a total of 35 first-year birds (17 birds in 2019 and 18 birds in 2020) to outdoor aviaries near the capture site. Each bird was randomly assigned to one of two roughly equal-sized housing groups (group A: 9 birds in 2019 and 9 in 2020; group B: 8 birds in 2019 and 9 in 2020). During the study periods (12 September–4 October 2019 and 10–22 September 2020), each housing group was kept together in one aviary equipped with perches, reeds, a water basin and food trays. Water and food [a mixture of live meal worms (*Tenebrio molitor*), dried insects and grated carrots] were provided *ad libitum*. The aviaries were made of non-magnetic materials (wood, polyester nets and insect mesh) and weakly magnetic materials (stainless steel screws) to minimise distortion of the Earth's magnetic field. Further, the aviaries gave the birds an unobstructed view of the surrounding habitat and access to various orientation cues (e.g. the sun and sun-related cues, the stars and local odours). At the end of the study period, i.e. before the end of the reed warbler's autumn migration season, all birds were released close to the capture site.

#### Experimental design and procedures

##### Experiment 1

**Fig. 1.**
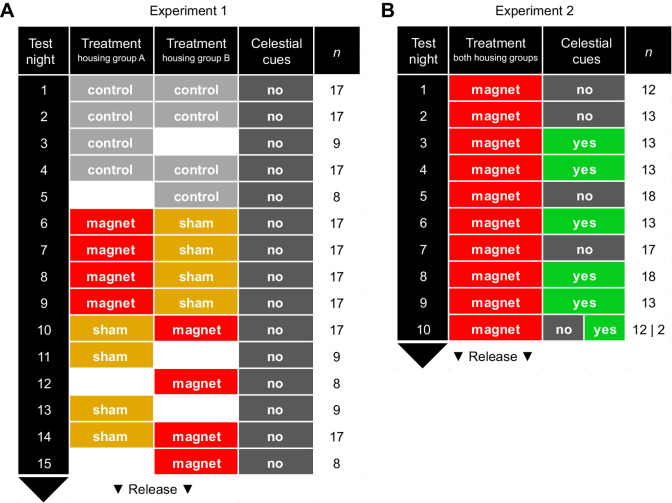
**Schematic overview of the experimental procedure during the repeated orientation tests of experiments 1 and 2.** (A) Experiment 1: each of the 17 birds included in the experiment underwent a total of 12 orientation tests within a test period of 15 nights, four orientation tests under each experimental treatment (control; magnet; sham). Birds in housing group A (9 birds) and housing group B (8 birds) were subjected to the magnet and sham treatment in opposing order and had no access to celestial cues in either test. (B) Experiment 2: each of the 18 birds included in the experiment underwent a total of 8 orientation tests within a test period of 10 nights, four orientation tests under each experimental treatment (magnet; magnet+celestial cues). Birds in both housing groups were subjected to the magnet treatment during the test period. Access to celestial cues during the orientation tests was given (yes, i.e. magnet+celestial cues) or denied (no) in alternating fashion among the test nights, depending on the local weather (cloud cover) during the test period. For both experiments, the number of birds tested each night of the test periods is given.

All 17 birds used for this experiment were repeatedly tested in Emlen funnels (see ‘Orientation tests’, below, for details) while being subjected to three different experimental treatments, i.e. control/no attachment, magnet attachment and sham attachment, during their autumn migration season in 2019. During the orientation tests, independent of the respective experimental treatment, birds were denied access to orientation cues other than the Earth's magnetic field (see ‘Orientation tests’, below, for details). Each bird underwent four orientation tests during each experimental treatment, for a total of 12 orientation tests on 12 different nights within the test period (15 test nights in total for experiment 1). All birds were tested in four control tests first. Subsequently, the treatment order differed between the two housing groups (group A and group B; see above). Birds assigned to group A were subjected to four tests during the magnet treatment, followed by four tests during the sham treatment. Birds assigned to group B were subjected to four tests during the sham treatment, followed by four tests during the magnet treatment (Fig. 1A). The rationale was to have a time-balanced design of the tests to exclude any temporal bias in the data (e.g. an ‘endogenously controlled change of migratory direction’ or ‘Zugknick’; Gwinner and Wiltschko, 1978; Liechti et al., 2012).

**Fig. 2.**
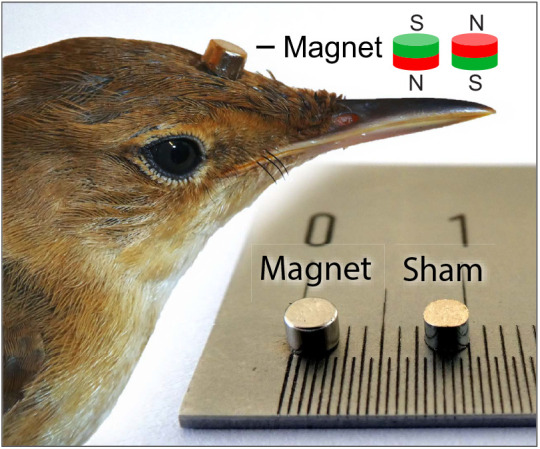
**Magnets and non-magnetic sham attachments used for the experiments.** Magnets/brass sham attachments were glued to the forehead of Eurasian reed warblers with their North Pole facing down/South pole facing up (experiment 1) or North Pole facing up/South pole facing down (experiment 2). Magnet and sham attachment dimensions: diameter 3 mm, height 2 mm; mass ca. 0.11 g.

During control tests, birds were tested with no attachments whatsoever in order to obtain their seasonally appropriate (control) magnetic compass orientation under exclusion of other orientation cues for subsequent comparisons. The lack of such a control orientation would render any following treatment difficult to interpret. For magnet and sham tests, we fitted a small magnet or a small non-magnetic sham attachment, respectively, to the birds’ forehead (Fig. 2). Both magnets and sham attachments were glued tightly to the short forehead plumage of the birds (Fig. 2) using super glue (LOCTITE^®^ Super Glue Gel). We used small (diameter: 3 mm, height: 2 mm, weight: 0.11 g) disc-shaped neodymium magnets (Supermagnete, S-03-02-N, EAN: 7640155436960, material: NdFeB, residual magnetism *B*_R_: 1.37–1.42 T, coercive field strength i*H*_C_: ≥955 kA m^−1^, energy product (*BH*)_max_: 358–382 kJ m^−^³). The magnets were attached with their North Pole facing down (towards the bird's skull)/South Pole facing up (away from the bird's skull; Fig. 2). As non-magnetic sham attachments, we used disc-shaped pieces cut from a brass rod, which resembled the magnets in dimensions and mass (Fig. 2). To habituate the birds to magnets and sham attachments, they were fitted during the night before the first magnet and sham test, respectively. If birds had lost their magnet or sham attachment during the day, these were replaced before the next orientation test (replacement required in 30% of the individual orientation tests).

##### Experiment 2

The 18 birds used for this experiment were repeatedly tested in Emlen funnels (see ‘Orientation tests’, below, for details) while being subjected to two different experimental treatments, i.e. magnet attachment and magnet attachment with celestial cues available (magnet+celestial cues) during their autumn migration season in 2020. During the orientation tests, the birds’ access to orientation cues other than the Earth's magnetic field depended on the respective experimental treatment (see ‘Orientation tests’, below). Each bird underwent four orientation tests during each experimental treatment, for a total of 8 orientation tests on 8 different nights within the test period (10 test nights in total for experiment 2). In contrast to experiment 1, birds from the two housing groups (group A and group B) were subjected to the same treatment in the same order during the whole test period. We started with magnet tests during the first test nights, which were then alternated with magnet+celestial cues tests during subsequent nights, depending on the local weather conditions and the availability of stars/absence of clouds during the test period. Orientation tests were conducted until each bird had been tested 4 times under each of the two experimental treatments (Fig. 1B).

For both magnet and magnet+celestial cues tests, we attached a small magnet to the bird’s forehead as in experiment 1. Dimensions and properties of the magnets were identical to those detailed above. Contrary to experiment 1, however, the magnets were attached with their North Pole facing up (away from the bird's skull)/South Pole facing down (towards the bird's skull; Fig. 2). The rationale was to gather additional information on whether a different orientation (i.e. a reversed polarity) of the attached magnet would result in an altered treatment effect. The attachment took place during the night before the first orientation test and magnets were replaced before the next orientation test if they got lost during the day (replacement required in 19% of the individual orientation tests).

#### Placement of the magnets

The sensory basis for magnetic compass orientation is the subject of active research and intense scientific debate (e.g. Mouritsen, 2015; Nordmann et al., 2017; Mouritsen, 2018). Several different models and hypotheses have been put forward to date, proposing very distinct avian magnetic senses that could be used to acquire directional (i.e. compass) information from Earth's magnetic field for orientation purposes (e.g. Mouritsen, 2015, 2018). The radical pair model, a leading concept for the magnetic compass of birds, proposes reversible light-dependent chemical reactions inside the retina of the birds’ eyes as the basis for the avian magnetic sense providing directional information, with the yield of these reactions depending on the alignment of a specific type of molecule (cryptochromes) to the magnetic field (e.g. Ritz et al., 2000; Hore and Mouritsen, 2016; Xu et al., 2021). The magnetite model, in contrast, proposes an avian magnetic sense based on biogenic magnetite (Fe_3_O_4_) structures located within nerve endings of the trigeminal nerve ophthalmic branch V1 in the birds’ upper beak (e.g. Kirschvink and Gould, 1981; Falkenberg et al., 2010; Heyers et al., 2010). Apart from these two models, an avian magnetic sense located within the semi-circular canals of the inner ear has been hypothesised, which might be based on electromagnetic induction (Nimpf et al., 2019).

We decided to fit magnets and sham attachments of our magnet, sham and magnet+celestial cues treatments to the foreheads of the birds. This way, magnets were located in close proximity (<2 cm) to any target tissues holding putative magnetoreceptors, regardless of whether these are actually found in the retina of the birds’ eyes, nerve endings in their upper beak or semi-circular canals of their inner ears. While conducting some example measurements at Bangor University, UK, we found that the small disc-shaped neodymium magnets we used (see ‘Experimental design and procedures’, above) increase the total intensity of the local magnetic field by ∼83,000 nT at a distance of 2 cm above their South Pole. It is worth mentioning, however, that the magnetic field induced by such neodymium magnets changes strongly and anisotropically as a function of both distance and direction from its centre. The total intensity of Earth's magnetic field shows a global range of approximately 25,000–65,000 nT (https://www.ngdc.noaa.gov/geomag/, accessed 1 June 2021), and amounts to approximately 48,800 nT (estimated for 17 September 2020; https://www.ngdc.noaa.gov/geomag/calculators/magcalc.shtml, accessed 1 June 2021) at our study site in Austria. This means that, during our magnet and magnet+celestial cues treatments, birds’ putative magnetoreceptors located in close proximity (<2 cm) to the attached magnets were exposed to a magnetic field with the total intensity increased by probably more than 120% of the natural global maximum. Further, putative magnetoreceptors that are situated in more than a single location would most probably be exposed to very different conditions as a result of the steep and anisotropic magnetic gradients induced by the attached magnet.

#### Orientation tests

Orientation tests lasted for 30 min each and were conducted in two successive sessions within the same night (one session for each of the housing groups, i.e. group A and group B), with the first session starting about 90 min after sunset (approximately at the end of the evening twilight period when the sun and sun-related cues are unavailable for orientation purposes). The assignment to the first and second session was alternated between the housing groups to allow a balanced experimental design and avoid any temporal bias in the data. We used modified Emlen funnels – the classical approach for testing migratory orientation in songbirds since its establishment by Emlen and Emlen (1966). The Emlen funnels were made of aluminium (top diameter: 350 mm, bottom diameter: 100 mm, slope 45 deg). Before the orientation tests, we placed up to nine Emlen funnels on a levelled wooden table (tabletop: ca. 1 m×1 m) set up under the open sky on a meadow at a distance of approximately 150 m to the Biological Station. We provided no artificial light during the orientation tests. Instead, we surrounded the table with vertical wooden panels (up to ca. 40 cm above the tabletop) to screen off any artificial light sources at the horizon (see fig. S2C of Kishkinev et al., 2021).

During all orientation tests of experiment 1 (control, magnet and sham) and during magnet orientation tests of experiment 2, the top of each Emlen funnel was covered with a translucent acrylic glass lid that served as a light diffuser and prevented the birds from seeing their surroundings and gathering information from orientation cues other than the local geomagnetic field. During the magnet+celestial cues orientation tests of experiment 2, however, the top of each Emlen funnel was covered with insect mesh that gave the birds an unobstructed view of the starry, clear (<50% cloud cover), moonless night sky and, thus, access to celestial orientation cues (i.e. stars) during the tests.

The directionality of the birds’ activity, i.e. their orientation, was recorded as their scratch marks left on a print film coated with a dried mixture of whitewash and glue. When an Emlen funnel is lined with such a print film, its two ends slightly overlap. During orientation tests, the alignment of the different funnels was alternated, with the overlapping point facing in different cardinal directions (i.e. north and south). The funnel alignment was unknown to the researchers who assessed the birds’ mean directions based on the distribution of the scratch marks from each orientation test. Instead, mean directions were estimated assuming an alignment to the North and later corrected according to the actual alignment from the record. This procedure was meant to avoid any observer bias with regard to directional assessment as well as to avoid providing unintentional visual cues to the birds. Two researchers (F.P. and F.B., F.P. and B.K. or F.P. and C.M.) independently assessed each bird's mean direction from the distribution of the scratch marks. At least one of the researchers was unaware of the respective experimental treatment during the directional assessment, except for magnet and sham tests of experiment 1 during which both researchers were unaware of the respective experimental treatment as these were conducted during the same period. The resultant direction from the two researchers’ recordings was taken into further analysis. If both researchers considered the scratch marks to be randomly distributed or their assessed directions deviated by more than 30 deg, a test was considered to be not oriented and, thus, discarded [24% (experiment 1) and 25% (experiment 2) of the orientation tests]. To ensure comparability with previous studies, only tests with at least 35 scratch marks (a common activity threshold; Wiltschko et al., 1998) and a clear unidirectional orientation were taken into analysis. Tests with fewer than 35 scratch marks were considered to reveal a lack of migratory activity and, thus, discarded [12% (experiment 1) and 8% (experiment 2) of the orientation tests]. Birds’ individual directions were used to calculate individual mean directions for each of the experimental treatments. From individual mean directions, group mean directions were calculated for the different experimental treatments of experiments 1 and 2.

### Statistics

Statistical analyses were conducted using the software R version 4.0.4 (http://www.R-project.org/).

**Fig. 3.**
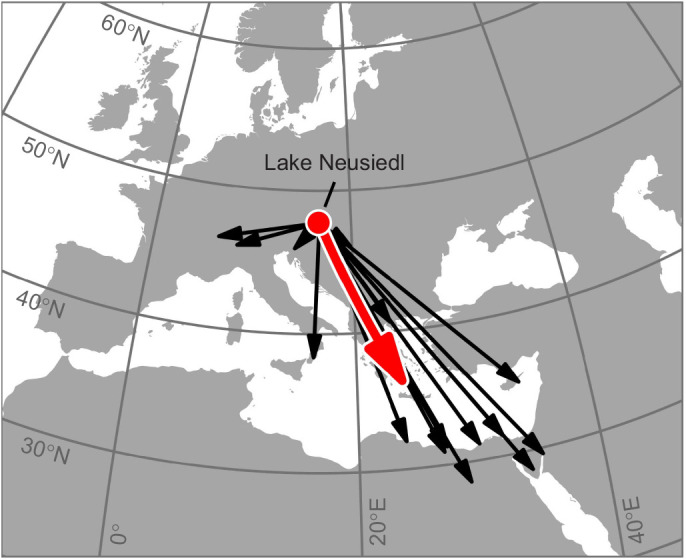
**Natural migratory direction of Eurasian reed warblers from Lake Neusiedl during autumn.** Migration direction was derived from recoveries of birds ringed at Lake Neusiedl (red dot) in both Austria and Hungary during summer and early autumn the same year. Black arrows depict great circle lines between the ringing site and the respective recovery sites (>250 km distance from ringing site); red arrow depicts the birds’ mean migratory direction and mean recovery distance based on these recoveries. Bird ring recovery data were provided by the Austrian Ornithological Centre and the Hungarian Bird Ringing Centre. The map is an orthographic projection with Lake Neusiedl as the projection centre.

We analysed bird ring recovery data to estimate the natural migratory direction of reed warblers from our study population during autumn migration. We used the package ‘geosphere’ (https://cran.r-project.org/web/packages/geosphere/index.html) to calculate bearings between the ringing sites (at Lake Neusiedl) and the respective recovery sites (Fig. 3). Then, we tested whether these bearings significantly differed from a uniform distribution using the Rayleigh test of uniformity and calculated their circular mean using the package ‘circular’ (http://cran.r-project.org/web/packages/circular/index.html). The circular mean was adopted as the study population's approximate natural migratory direction.

To test whether the orientation data obtained during the different experimental treatments of experiments 1 and 2 significantly differed from a uniform distribution, the Rayleigh test of uniformity was used. Additionally, we assessed the likelihood of the 10 models for orientation behaviour described by Schnute and Groot (1992) for the orientation data obtained during each experimental treatment of experiments 1 and 2 using the model selection procedure implemented in the package ‘CircMLE’ (Fitak and Johnsen, 2017). These comprise uniform (M1), unimodal (M2A), symmetric modified unimodal (M2B), modified unimodal (M2C), homogenous symmetric bimodal (M3A), symmetric bimodal (M3B), homogenous axial bimodal (M4A), axial bimodal (M4B), homogenous bimodal (M5A) and bimodal (M5B) orientation models. We compared the models by means of the corrected Akaike information criterion (AIC_c_; Hurvich and Tsai, 1989) and the corresponding AIC_c_ model weights.

In order to compare orientation data between the experimental treatments during which birds were significantly oriented (according to the Rayleigh test of uniformity), we used the non-parametric Mardia–Watson–Wheeler test implemented in the package ‘circular’ (http://cran.r-project.org/web/packages/circular/index.html). For comparisons of orientation data between the experimental treatments during which birds were not significantly oriented with those during which they were significantly oriented, we followed a bootstrap approach applied by Chernetsov et al. (2017). This approach uses the mean resultant vectors (*r*-values; a measure of directedness) obtained during the different experimental treatments and compares whether the *r*-value that derives from a not significantly oriented sample falls within the same confidence intervals (CI) for another *r*-value that derives from a significantly oriented sample. In a first step, a random sample of *n* orientation directions is drawn with replacement from the original (significantly oriented) sample of *n* orientation angles obtained during the respective experimental treatment (e.g. *n*=17 for the control tests) and the corresponding *r*-value is calculated. This procedure is repeated 100,000 times, each time with a new randomisation. In a second step, the resulting 100,000 *r*-values are ranked in ascending order, with values at the ranks 2500 and 97,500, 500 and 99,500, and 50 and 99,950 defining the 95%, 99% and 99.9% confidence limits for the observed *r*-value of the significantly oriented sample, respectively. If the observed *r*-value of the not significantly oriented sample is outside these CI, the significantly oriented sample is more directed with a confidence of >95%, >99% and >99.9%, respectively.

## RESULTS

### Natural migratory direction

Autumn bird ring recoveries of reed warblers ringed at Lake Neusiedl suggested a natural migratory direction towards the SE to SSE [mean direction: α=152 deg (all directions are indicated relative to magnetic North); Rayleigh test: *r*=0.80, *P*<0.001, *n*=19; 95% CI of the group mean direction 140–170 deg; Fig. 3].

### Orientation experiments

#### Experiment 1

**Fig. 4.**
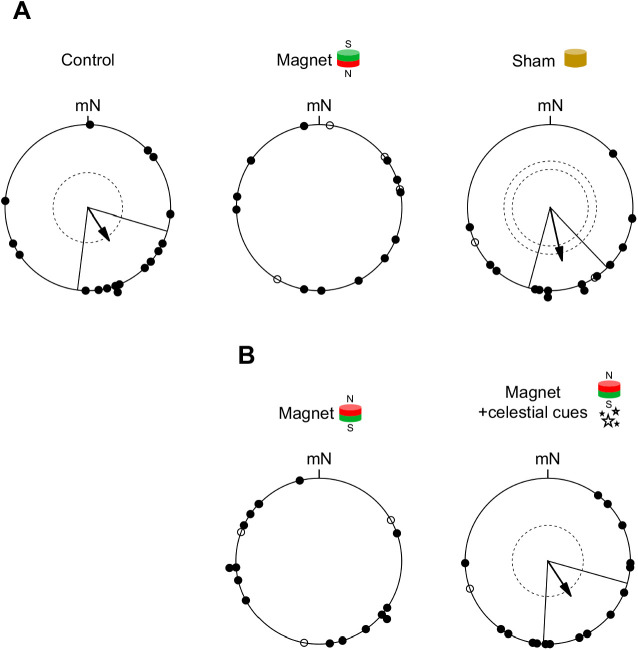
**Results from the orientation tests of experiments 1 and 2.** (A) Magnetic compass orientation of first-year Eurasian reed warblers tested under the different experimental treatments (control, magnet, sham) of experiment 1 during autumn migration. (B) Magnetic and celestial compass orientation of first-year Eurasian reed warblers tested under the different experimental treatments (magnet, magnet+celestial cues) of experiment 2 during autumn migration. Circular diagrams depict the birds’ individual mean directions from repeated orientation tests (filled circles). Birds that showed directionality only once during repeated orientation tests (open circles) were not considered in subsequent analyses. Arrows show the group-specific mean directions and the mean resultant vector lengths of significantly oriented treatment groups (according to the Rayleigh test of uniformity); dashed circles indicate the radius the respective mean resultant vector needs for the 5% and 1% significance levels according to the Rayleigh test of uniformity; solid lines flanking the arrows give the 95% confidence intervals for the group-specific mean directions; all directions are depicted relative to magnetic North (mN).

We repeatedly tested a total of 17 first-year reed warblers for their magnetic compass orientation while they were subjected to three different experimental treatments (control, magnet, sham) during the autumn migration season. The circular distributions obtained were best described by unimodal orientation models (Table 1) and birds showed a mean orientation towards the SE to SSE during both control tests (mean direction: α=147 deg; Rayleigh test: *r*=0.47, *P*=0.02, *n*=17; 95% CI mean=105–187 deg; Fig. 4A) and sham tests (mean direction: α=167 deg; Rayleigh test: *r*=0.65, *P*=0.002, *n*=14; 95% CI mean=136–195 deg; Fig. 4A). The two circular distributions were not found to be statistically distinguishable (Mardia–Watson–Wheeler test: *W*=0.56, *P*=0.76). During magnet tests, the birds were randomly oriented (Rayleigh test: *r*=0.17, *P*=0.70, *n*=12; Fig. 4A) and their circular distribution was best described by the uniform orientation model (Table 1). This apparent lack of a mean orientation was distinguishable from the orientation of the same birds during both control tests (>95% confidence: the bootstrapped 95% CI for the *r*-value from control tests was 0.21<*r*<0.76, which does not overlap with the *r*-value of 0.17 obtained during magnet tests) and sham tests (>99.9% confidence: the bootstrapped 99.9% CI for the *r*-value from sham tests was 0.30<*r*<0.93, which does not overlap with the *r*-value of 0.17 obtained during magnet tests).

**Table 1.**
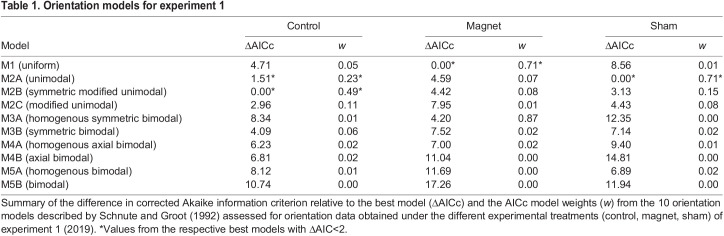
Orientation models for experiment 1

#### Experiment 2

We repeatedly tested a total of 18 first-year reed warblers for their magnetic and celestial compass orientation while they were subjected to two different experimental treatments (magnet, magnet+celestial cues) during the autumn migration season. During magnet tests, the birds were not oriented in a unimodal way (Rayleigh test: *r*=0.23, *P*=0.48, *n*=15; Fig. 4B) and their circular distribution was best described by either the homogenous symmetric bimodal orientation model or the uniform orientation model (Table 2). The circular distribution obtained during magnet+celestial cues tests was best described by a unimodal orientation model (Table 2) and birds showed a mean orientation towards the SE to SSE (mean direction: α=146 deg; Rayleigh test: *r*=0.51, *P*=0.01, *n*=16; 95% CI mean=104–184 deg; Fig. 4B). The lack of a unimodal mean orientation during magnet tests was distinguishable from the orientation of the same birds during magnet+celestial cues tests (>95% confidence: the bootstrapped 95% CI for the *r*-value from magnet+celestial cues was 0.30<*r*<0.76, which does not overlap with the *r*-value of 0.23 obtained during magnet tests).

**Table 2.**
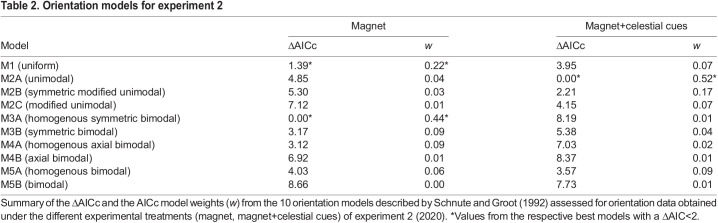
Orientation models for experiment 2

## DISCUSSION

In this study, we show the disruptive effect of small magnets on magnetic compass orientation in a migratory songbird, the reed warbler, under controlled experimental conditions. During control and sham tests, i.e. when Earth's magnetic field was available as the sole orientation cue, reed warblers were oriented towards the SE to SSE (Fig. 4A), matching the seasonally appropriate, natural, migratory direction of the study population during autumn migration (Fig. 3). During magnet tests, however, when birds had small magnets attached to their foreheads intended to prevent them from gathering meaningful magnetic information, birds appeared disoriented, with the obtained orientation data being best described either by a uniform distribution (Fig. 4A, Table 1) or by symmetric bimodal and uniform distributions (Fig. 4B, Table 2). When provided with an unobstructed view of the starry night sky during magnet+celestial cues tests, in contrast, the birds were able to orient in the seasonally appropriate, natural, migratory direction towards the SE to SSE, despite having small magnets attached to their foreheads (Fig. 4B). This suggests that the birds can fall back on other orientation cues if they find Earth’s magnetic field to be unusable for determining direction.

Magnetic compass orientation has repeatedly been reported in various songbird species and is now well established for this taxon (e.g. Mouritsen, 2015; Chernetsov, 2017). Furthermore, there is evidence for songbirds using the Earth's magnetic field as a source of not only directional (i.e. compass) information but also positional (i.e. map) information during the navigation process (e.g. Kishkinev et al., 2013, 2015; Chernetsov et al., 2017; Pakhomov et al., 2018; Kishkinev et al., 2021). With regard to other bird taxa that include larger species, however, evidence for the use of Earth's magnetic field for orientation or navigation appears less coherent. Although some experimental studies have shown magnetic compass orientation in homing pigeons (*Columba livia domestica*) (e.g. Keeton, 1969, 1971; Walcott and Green, 1974; Ioalè, 1984, 2000; Visalberghi and Alleva, 1979; Gagliardo et al., 2009; Mora and Walker, 2012), others criticised these studies and/or failed to replicate the prior results (e.g. Lamotte, 1974; Moore, 1988; Ranvaud et al., 1991; reviewed in Wiltschko and Wiltschko, 1995a; Wallraff, 2005). For gulls (Laridae), there is evidence for magnetic compass orientation from displacement experiments with fledglings (e.g. Southern, 1972), but the significance of magnetic information for their navigational performance has been questioned based on data from an experimental study tracking long-distance flight behaviour of gulls by means of satellite telemetry (Wikelski et al., 2015). The latter holds true for tubenoses (Procellariiformes) as well, which to the best of our knowledge have not been shown to use Earth's magnetic field to obtain directional (i.e. compass) or positional (i.e. map) information for orientation and navigation during their offshore flights in any experimental study yet (e.g. Massa et al., 1991; Mouritsen et al., 2003; Benhamou et al., 2003; Bonadonna et al., 2003, 2005; Gagliardo et al., 2013; Pollonara et al., 2015; Padget et al., 2017; Syposz et al., 2021). A recent correlational study, however, suggests that the inclination of Earth's magnetic field plays a role for finding and selecting breeding colonies during the recruitment phase in a tubenose species (Wynn et al., 2020).

The vast majority of studies that aimed to investigate the use and overall significance of magnetic information for orientation and navigation in ‘non-passerine’ birds and in the context of free-flight behaviour used rare-earth magnets in a way comparable to our current study (e.g. Wiltschko and Wiltschko, 1995a; Mouritsen et al., 2003). Hence, we expected that our results would be comparable with those of the previous studies and could help to put them into context. Generally, it can be stated that studies which found a disruptive effect of the magnets on birds’ orientation behaviour were usually able to largely exclude orientation cues other than the Earth's magnetic field during the experiments (i.e. complete overcast conditions). Studies in which the birds apparently had access to other (e.g. celestial) orientation cues (i.e. clear skies), however, show a tendency towards finding no effect of the magnets on the orientation behaviour and overall navigation performance (e.g. Keeton, 1969, 1971; Ioalè, 1984, 2000; Mouritsen et al., 2003; Bonadonna et al., 2005; Gagliardo et al., 2013; Pollonara et al., 2015; Padget et al., 2017; but see Southern, 1972; reviewed in Wiltschko and Wiltschko, 1995a; Wallraff, 2005). This is in general agreement with the results of the current study. The magnets attached to the birds’ foreheads were found to disrupt their magnetic compass orientation, but the disruptive effect was not detectable when the birds had access to celestial orientation cues, which apparently gave them the opportunity to fall back on another compass system for successful orientation. If orientation cues are accessible, birds may integrate all their different compass systems (magnetic and celestial compasses; Wiltschko and Wiltschko, 2001), but for successful orientation they seem to require merely one at a time (e.g. Mouritsen, 2015). Impaired access to celestial orientation cues may occur under overcast conditions, and access to Earth's magnetic field for orientation and navigation may get severely impacted by both natural (e.g. solar storms; Bianco et al., 2019) and anthropogenic (e.g. electromagnetic noise; Engels et al., 2014) causes that birds must cope with. Hence, independent of any underlying compass system hierarchy and calibration (e.g. Muheim et al., 2006; Liu and Chernetsov, 2012; Pakhomov and Chernetsov, 2020), birds need their compass integration mechanisms to allow reversion to the other systems, if one becomes temporarily inaccessible or unreliable. This is supported by both the current results and previous studies (e.g. Wiltschko et al., 1987; Mouritsen, 1998).

Our study species, the reed warbler, represents a well-established model for migratory songbirds and has repeatedly been shown to use Earth's magnetic field for orientation and navigation in a migratory context (e.g. Holland, 2010; Kishkinev, et al., 2015; Chernetsov et al., 2017; Kishkinev et al., 2021). Thus, by studying reed warblers in orientation cages under controlled experimental conditions, we are able to provide clear evidence for the general efficacy of the magnet approach for temporarily disrupting magnetic compass orientation in birds. Further, the efficacy of the magnet approach appears independent of the relative alignment of the magnets in our orientation experiments (i.e. North Pole facing down/South pole facing up in experiment 1 versus North Pole facing up/South pole facing down in experiment 2). Studies that applied the magnet approach to investigate the significance of Earth's magnetic field for orientation and overall navigation performance of birds under free-flight conditions have previously been criticised for the potential inefficacy of the experimental treatment (e.g. Wang et al., 2006), which is understandable given that many of them did not find a disruptive effect. With regard to our current results, however, we argue that this general criticism is no longer tenable. Instead, we would like to emphasise that the detectability of a disruptive effect of the magnets appears highly context dependent. This is crucial for the interpretation of results from studies following the magnet approach under free-flight conditions, which usually do not allow the restriction of birds’ access to other cues for orientation and navigation.
